# Supplementary material for: Socio-ecological correlates of physical activity in breast and colon cancer survivors 4 years after participation in a randomized controlled exercise trial (PACT study)
Source: PLoS One. 2020 Apr 16;15(4):e0231663. doi: 10.1371/journal.pone.0231663 (PMC7161977; doi:10.1371/journal.pone.0231663)
Supplement: S1 Table — (DOC) [file pone.0231663.s001.doc]

| **S1 Table.**  Univariable associations between candidate social-ecological correlates and moderate-to-vigorous total physical activity levels (min/week) and moderate-to-vigorous sport and leisure PA (min/week). | | | | | | | | | | | | | | | | | | |
| --- | --- | --- | --- | --- | --- | --- | --- | --- | --- | --- | --- | --- | --- | --- | --- | --- | --- | --- |
|  | **Moderate-to-vigorous Total PA** | | | | | | | | | **Moderate-to-vigorous Leisure and Sport PA** | | | | | | | | |
|  | Baseline | | | Change during intervention | | | Follow-up at 4 yrs | | | Baseline | | | Change during intervention | | | Follow-up at 4 yrs | | |
|  | 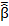 | | *p* | | 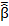 | *p* | | 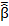 | *p* | | 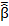 | *p* | | 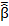 | *p* | | 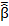 | *p* |
| **Socio-demographical characteristics** | | | | | | | | | | | | | | | | | | |
| Age | -0.02 | | 0.41 | | ● | ● | | -0.02 | 0.28 | | -0.10 | 0.24 | | ● | ● | | -0.10 | 0.24 |
| Marital status | 0.48 | | 0.28 | | ● | ● | | 0.16 | 0.69 | | 0.97 | 0.61 | | ● | ● | | 0.80 | 0.66 |
| Employment | ● | | ● | | ● | ● | | 0.16 | 0.65 | | ● | ● | | ● | ● | | 0.30 | 0.84 |
| Education level | 0.42 | | 0.49 | | ● | ● | | ● | ● | | **5.26** | **0.04** | | ● | ● | | ● | ● |
| BMI | 0.005 | | 0.89 | | 0.02 | 0.62 | | 0.02 | 0.66 | | **-0.30** | **0.08** | | -0.12 | 0.45 | | **-0.32** | **0.07** |
| **Clinical characteristics** | | | | | | | | | | | | | | | | | | |
| Tumor site | -0.28 | | 0.55 | | ● | ● | | ● | ● | | -2.04 | 0.31 | | ● | ● | | ● | ● |
| Radiotherapy | 0.05 | | 0.89 | | ● | ● | | ● | ● | | 0.57 | 0.69 | | ● | ● | | ● | ● |
| **Physical characteristics** | | | | | | | | | | | | | | | | | | |
| VO_2_ _peak_ (L/min) | **0.67** | | **0.07** | | 0.001 | 0.95 | | ● | ● | | **3.89** | **0.02** | | -0.04 | 0.37 | | ● | ● |
| Handgrip strength (KgF) | 0.02 | | 0.29 | | -0.02 | 0.25 | | ● | ● | | 0.03 | 0.76 | | -0.03 | 0.61 | | ● | ● |
| Extensor peak torque at 60/s (Nm) | <-0.001 | | 0.92 | | -0.003 | 0.59 | | ● | ● | | 0.003 | 0.89 | | -0.02 | 0.33 | | ● | ● |
| Baseline total PA | **0.03^b^** | | **<0.001** | | <0.001 | 0.65 | | ● | ● | | **0.08^b^** | **0.002** | | <0.001 | 0.68 | | ● | ● |
| Baseline leisure and sport PA | **0.003** | | **<0.001** | | <0.001 | 0.44 | | ● | ● | | **0.01** | **<0.001** | | <-0.001 | 0.77 | | ● | ● |
| **Environmental characteristics** | | | | | | | | | | | | | | | | | | |
| Private recreation facilities <1km | | ● | ● | | ● | ● | | 0.004 | 0.84 | | ● | ● | | ● | ● | | **2.56^a^** | **0.001** |
| Private recreation facilities <5km | | ● | ● | | ● | ● | | 0.0004 | 0.82 | | ● | ● | | ● | ● | | **2.32^a^** | **0.02** |
| Total green and open space <1km | | ● | ● | | ● | ● | | **-0.25^a^** | **0.19** | | ● | ● | | ● | ● | | 0.85^a^ | 0.30 |
| Total green and open space <5km | | ● | ● | | ● | ● | | **-0.39^a^** | **0.08** | | ● | ● | | ● | ● | | -0.52^a^ | 0.79 |
| Residential area <1km | | ● | ● | | ● | ● | | 0.004 | 0.64 | | ● | ● | | ● | ● | | **0.10** | **0.005** |
| Residential area <5km | | ● | ● | | ● | ● | | -0.002 | 0.93 | | ● | ● | | ● | ● | | **0.22** | **0.02** |
|  | | | | | | | | | | | | | | | | | | |
| **Psychosocial characteristics – MFI** | | | | | | | | | | | | | | | | | | |
| General fatigue | **-0.09** | | **0.02** | | **0.005** | **0.09** | | **-0.08** | **0.05** | | **-0.41** | **0.02** | | 0.01 | 0.27 | | **-4.30^a^** | **0.01** |
| Physical fatigue | **-0.14** | | **0.0006** | | **0.004** | **0.06** | | **-0.09** | **0.02** | | **-0.55** | **0.001** | | **0.02** | **0.07** | | **-5.32^a^** | **0.001** |
| Reduced activity | **-0.15** | | **0.0006** | | 0.003 | 0.27 | | **-0.13** | **<0.001** | | **-5.84^a^** | **0.004** | | 0.01 | 0.28 | | **-0.56** | **0.001** |
| **Psychosocial characteristics - EORTC QLQ-C30** | | | | | | | | | | | | | | | | | | |
| Global health status | 0.003 | | 0.76 | | 0.004 | 0.33 | | **0.02** | **0.06** | | 0.04 | 0.35 | | -0.006 | 0.70 | | **0.09** | **0.03** |
| Physical functioning | **0.03** | | **0.04** | | **0.009** | **0.19** | | **0.03** | **0.02** | | 0.06 | 0.27 | | 0.03 | 0.23 | | **0.18** | **0.001** |
| Emotional functioning | **0.02** | | **0.11** | | 0.007 | 0.33 | | **0.01** | **0.08** | | 0.02 | 0.65 | | 0.02 | 0.56 | | 0.02 | 0.63 |
| Social functioning | -0.0001 | | 0.99 | | 0.0002 | 0.50 | | **0.01** | **0.19** | | 0.02 | 0.59 | | <0.001 | 0.75 | | 0.02 | 0.70 |
| Pain | -0.005 | | 0.53 | | -0.0001 | 0.57 | | -0.005 | 0.50 | | -0.02 | 0.59 | | <-0.001 | 0.74 | | **-0.05** | **0.13** |
| HADS | **-0.05** | | **0.12** | | -0.0004 | 0.67 | | ● | ● | | -0.07 | 0.94 | | -0.003^a^ | 0.44 | | ● | ● |
| **Randomization^c^** | -0.17 | | 0.61 | | ● | ● | | ● | ● | | -0.72 | 0.61 | | ● | ● | | ● | ● |

*Abbreviations:* *PA* physical activity, *BMI* body mass index, *MFI* multifactorial fatigue inventory, *HADs* hospital anxiety and depression scale. The variables that belong to the values in **bold** were selected as relevant correlate of PA. ^a^These correlates were log-transformed in order to meet the linearity assumption. ^b^These correlates were transformed using the box-cox method in order to meet the linearity assumption. ^c^In contrast to our previously published paper (Witlox et al. (2018)), randomization was not a significant correlate of PA. This can be attributed to the fact that we only included participants who participated in the 4 year post-baseline measurements instead of all participants of the PACT study. Besides, we did not control for baseline values of PA in the current analyses.
